# Bisoprolol and Amlodipine Co-Administration with Glimepiride in a Diabetic Rat Model: A Statistical and Machine Learning Analysis

**DOI:** 10.3390/ph19071064

**Published:** 2026-07-10

**Authors:** Mohammad Hailat, Zeyad Hailat, Mo’ath Ifraitekh, Zainab Zakaraya, Marwan Shalash, Israa Al-Ani, Wael Abu Dayyih

**Affiliations:** 1 Faculty of Pharmacy, Al-Zaytoonah University of Jordan, Amman 11733, Jordan; m.hailat@zuj.edu.jo; 2 Department of Information Systems, Yarmouk University, Irbid 21163, Jordan; zeyad.hailat@yu.edu.jo; 3 Faculty of Pharmacy and Medical Sciences, University of Petra, Amman 11196, Jordan; muath.ifraitekh@gmail.com; 4 Department of Biopharmaceutics and Clinical Pharmacy, Faculty of Pharmacy, Al-Ahliyyah Amman University, Amman 11931, Jordan; z.zakaraya@ammanu.edu.jo; 5 Department of Pharmaceutics and Pharmaceutical Technology, Faculty of Pharmacy, Zarqa University, Zarqa 13111, Jordan; mshalash@zu.edu.jo; 6 Department of Pharmacy, Faculty of Pharmacy, Middle East University, Amman 11831, Jordan; i.alani@meu.edu.jo; 7 Department of Pharmaceutical Chemistry, Faculty of Pharmacy, Mutah University, Al Karak 61710, Jordan

**Keywords:** amlodipine, antihypertensive therapy, bisoprolol, drug–drug interaction, glimepiride, glycated hemoglobin, machine learning, preclinical pharmacology, streptozotocin, type 2 diabetes mellitus

## Abstract

**Background/Objectives**: Diabetes mellitus type 2 (T2DM) is often associated with hypertension, necessitating treatment with combinations of medications that address both glycemic control and blood pressure. Whether commonly co-prescribed antihypertensives modify the glycemic efficacy of a sulfonylurea remains insufficiently characterized in controlled preclinical models. **Methods**: One hundred adult male Wistar rats were allocated to ten parallel groups (*n* = 10): healthy and diabetic untreated controls; glimepiride, bisoprolol or amlodipine monotherapy (in healthy and diabetic animals); and the diabetic combinations glimepiride+bisoprolol and glimepiride+amlodipine. T2DM was induced with a high-fat diet plus low-dose streptozotocin (35 mg/kg, i.p.) and confirmed by fasting blood glucose ≥ 200 mg/dL. Glycated hemoglobin (HbA1c) was measured weekly for 11 weeks. Non-parametric inference (Kruskal–Wallis, Dunn’s with Bonferroni correction, Mann–Whitney U, Wilcoxon signed-rank) was complemented by Random Forest regression and PCA/K-means clustering. **Results**: Week-11 HbA1c differed markedly across groups (Kruskal–Wallis H = 94.3, *p* < 0.001). Glimepiride + bisoprolol achieved near-normal control (4.37% ± 0.15), statistically indistinguishable from healthy groups (*p* ≥ 0.33), and was the only diabetic regimen with a declining trajectory (−0.66 percentage points; Wilcoxon *p* = 0.004). Adding either antihypertensive to glimepiride did not worsen glycemic control. Amlodipine monotherapy did not attenuate hyperglycemia (8.47% ± 0.20), approaching that of untreated diabetic controls (9.31% ± 0.18), consistent with the absence of intrinsic glucose-lowering activity. All agents showed pronounced disease-state dependence (healthy–diabetic divergence 2.33–3.13 points). Random Forest prediction was accurate (R^2^ = 0.985), and unsupervised clustering separated effective from ineffective regimens, corroborating the statistical findings. **Conclusions**: In this model, bisoprolol co-administration enhanced and amlodipine co-administration preserved glimepiride-mediated glycemic control. Glimepiride+bisoprolol emerged as the most effective regimen, supporting cardioselective β-blockade as a metabolically favorable antihypertensive partner for sulfonylurea therapy and warranting clinical confirmation. More broadly, these results provide a preclinical, evidence-based rationale for selecting metabolically favorable antihypertensives in patients with coexisting T2DM and hypertension, with the potential to improve glycemic outcomes and reduce the risk of adverse drug–disease interactions during combination therapy.

## 1. Introduction

Hypertension and type 2 diabetes mellitus (T2DM) are among the most prevalent chronic conditions worldwide and co-occur far more often than would be expected by chance, reflecting a shared pathophysiology that includes insulin resistance, endothelial dysfunction, heightened sympathetic tone and dysregulation of the renin–angiotensin–aldosterone system [1,2]. Because the two diseases reinforce one another’s cardiovascular and metabolic risk, the majority of patients with T2DM ultimately require concurrent antihyperglycemic and antihypertensive pharmacotherapy [2,3]. In real-world practice, this dual burden is a major driver of polypharmacy and medication-related problems; in a large Jordanian study of refugees with chronic disease, hypertension and type 2 diabetes were the two most prevalent diagnoses, and drug therapy problems were highly prevalent [4]. Contemporary standards of care therefore call for individualized, comorbidity-aware selection of antihyperglycemic and antihypertensive therapy [5].

Selecting an antihypertensive agent for a patient with diabetes is not metabolically neutral. Classical, non-selective β-adrenergic blockers have historically been associated with impaired insulin sensitivity, blunted hypoglycemia awareness, and adverse effects on glucose handling. In contrast, newer cardioselective and vasodilating agents appear to be more metabolically favorable [6,7]. The calcium channel blocker (CCB) class (e.g., amlodipine) is generally considered a glucose-neutral agent, but evidence regarding interactions with sulfonylureas is inconsistent [8]. The clinical significance of these uncertainties lies in the fact that the oral hypoglycemic agent may, in theory, be either enhanced or reduced by the antihypertensive taken.

Glimepiride, a second-generation sulfonylurea, can reduce blood glucose levels, mainly because it stimulates the secretion of insulin from the pancreas, and is one of the most commonly used oral antidiabetic medications [9]; its pharmacokinetics and glycemic effect have been characterized in rodent models [10], and it is frequently co-administered with other antihyperglycemic agents in combinations that require validated, stability-indicating quantification [11,12]. Bisoprolol is a highly β1-selective β-blocker used in the treatment of hypertension and heart failure [13], and amlodipine is a long-acting dihydropyridine CCB with peripheral vasodilatory activity [6,8]. Preclinical data have been systematically gathered; however, descriptions of how each antihypertensive alters glycemic control via the integrative biomarker, followed longitudinally across distinct disease states, remain limited [14].

To address this void, an 11-week controlled study was conducted in 100 male Wistar rats in ten groups, which included untreated healthy and diabetic rats, each drug being used as single therapy in healthy and diabetic rats, and two combination therapies of glimepiride+bisoprolol and glimepiride+amlodipine in diabetic rats, using the high-fat diet/low-dose streptozotocin model of T2DM [15] that has been applied previously to evaluate adjunctive and combination antihyperglycemic interventions through longitudinal glyaemic monitoring [16,17,18]. The primary outcome was HbA1c levels measured weekly, a validated integrative marker of glycemic control [19]. To obtain the best information from the longitudinal data, we used traditional nonparametric biostatistics, along with supervised machine learning (Random Forest regression) and unsupervised clustering (principal component analysis with K-means) [20,21], to validate the main results with orthogonal methods of analysis. We specifically addressed four questions: (i) how bisoprolol and amlodipine influence longitudinal glycemic parameters and treatment-response patterns in glimepiride-treated diabetic rats; (ii) whether pharmacodynamically meaningful interactions between glimepiride and either antihypertensive affect blood glucose regulation; (iii) whether the addition of bisoprolol or amlodipine alters the antihyperglycemic efficacy of glimepiride; (iv) whether machine learning models can accurately identify and predict differences in glycemic outcomes among glimepiride, glimepiride–bisoprolol, and glimepiride–amlodipine treatment groups.

## 2. Results

### 2.1. Establishment of the Diabetic Model and Body-Weight Response

High-fat feeding followed by low-dose streptozotocin (STZ) resulted in persistent hyperglycemia in all diabetic groups (fasting blood glucose > 200 mg/dL after 3 weeks) and increased HbA1c levels compared with healthy animals, demonstrating the induction of a T2DM-like diabetic phenotype. Body weight was significantly different among disease states (Table 1). Weight gain in healthy animals occurred during the study period, whereas weight loss was progressive in untreated diabetic animals (from 260.4 ± 11.6 g at week 1 to 103.9 ± 5.7 g at week 11). Diabetic animals given a glimepiride-containing regimen were partially protected from this catabolic weight loss (endpoint weights ≈ 195–202 g), which was commensurate with their improved metabolic control.

### 2.2. Endpoint (Week-11) HbA1c: A Clear Treatment Hierarchy

**Figure 1:**
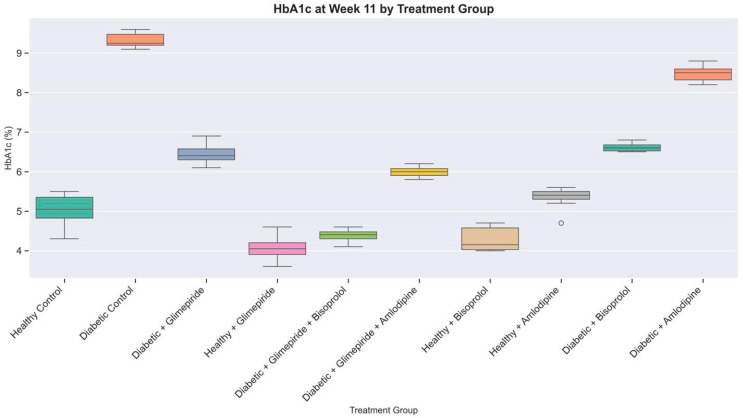
The distribution of HbA1c levels by week-11 for the 10 groups. Boxes represent the interquartile range, and the median line is drawn through the middle of the range. Whiskers reach 1.5 × IQR. The differences between the countries were highly significant (Kruskal–Wallis H = 94.31, *p* < 10^−9^). Glimepiride+bisoprolol managed to normalize the levels, while amlodipine monotherapy and the diabetic control group had persistently high levels.

Week-11 HbA1c spanned 4.94 percentage points across groups. It separated into three tiers (Table 2, Figure 1): near-normal control (glimepiride+bisoprolol, 4.37% ± 0.15, comparable to the healthy range of 4.04–5.34%); intermediate control (glimepiride+amlodipine 5.99% ± 0.12, glimepiride 6.44% ± 0.27 and bisoprolol 6.61% ± 0.10); and poor control (amlodipine 8.47% ± 0.20 and untreated diabetic control 9.31% ± 0.18). The within-group standard deviations were all quite low (0.10–0.42), and the medians were similar to the means, suggesting that the responses were consistent across animals rather than averaged across responders vs. non-responders.

### 2.3. Global and Pairwise Comparisons

The Shapiro–Wilk test indicated that the data were approximately normally distributed for 8 of the 10 groups. In contrast, Levene’s test revealed a lack of homogeneity of variance (L = 2.94, *p* = 0.004), suggesting the use of a nonparametric approach. The Kruskal–Wallis test confirmed highly significant global heterogeneity (H = 94.31, *p* < 10^−9^). Dunn’s Bonferroni-corrected post hoc comparisons (Table 3) showed that glimepiride+bisoprolol differed significantly from every diabetic group (*p* ≤ 0.015) yet was statistically equivalent to all healthy groups (*p* ≥ 0.328), confirming near-complete restoration of glycemic status. Amlodipine monotherapy did not differ significantly from the untreated diabetic control group (*p* = 1.00), indicating no glycemic benefit.

### 2.4. Longitudinal Trajectories and the Effect of Combination Therapy

**Figure 2:**
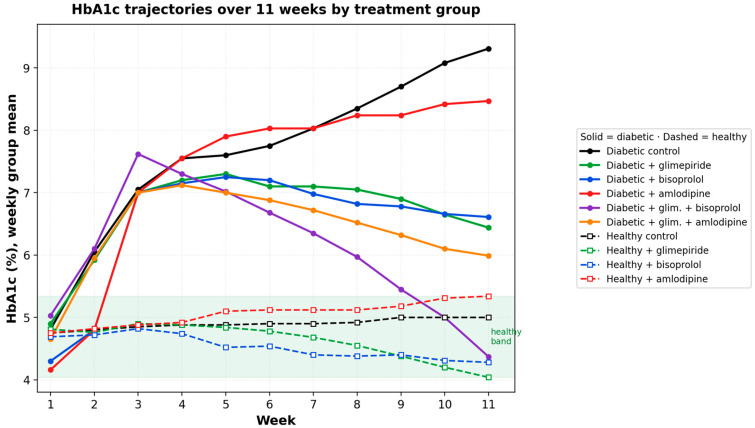
Weekly HbA1c trajectories (group means) over 11 weeks. Lines are colored by drug and styled by disease state (solid lines/filled markers = diabetic; dashed lines/open markers = healthy); the shaded band denotes the healthy HbA1c range (4.04–5.34%). Glimepiride+bisoprolol (purple) is the only diabetic regimen that declines toward the healthy band, whereas the untreated diabetic control (black) and amlodipine monotherapy (red) rise most steeply. Axes: Week (1–11) versus HbA1c (%).

**Figure 3:**
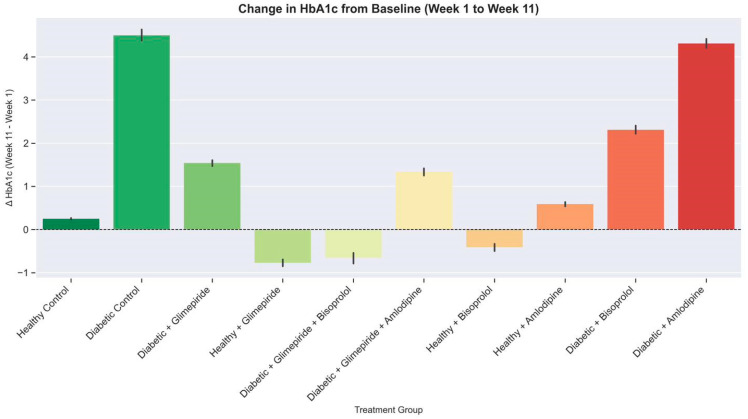
Change in HbA1c from baseline to week 11 by group, emphasizing the unique downward shift of glimepiride+bisoprolol against the upward shifts of all other diabetic regimens.

Tracking HbA1c weekly revealed a striking directional dichotomy (Figure 2 and Figure 3, Table 4). Within the diabetic cohorts, glimepiride+bisoprolol alone had a decreasing trend (−0.66 percentage points from baseline to week 11; Wilcoxon *p* = 0.004) and was trending towards healthy levels. On all other diabetic regimens, there was an increase over time: glimepiride+amlodipine increased by +1.34 points, glimepiride increased by +1.54 points, bisoprolol increased by +2.31 points, amlodipine increased by +4.31 points, and untreated control increased by +4.50 points (all Wilcoxon *p* ≤ 0.002). All animals receiving glimepiride+bisoprolol improved or stabilized, whereas those not receiving it deteriorated, underscoring the consistency of the pharmacodynamic effects.

Most importantly, there was no deleterious effect of either of the antihypertensives combined with glimepiride compared with using glimepiride alone in terms of glycemic control. Co-administration of bisoprolol resulted in a greater additive benefit than glimepiride alone (2.07 points; Dunn’s *p* = 1.7 × 10^−4^), suggesting a synergistic interaction between the drugs. In contrast, co-administration of amlodipine did not (0.45 points, Dunn’s *p* = 5.3 × 10^−4^). The word synergy is used descriptively because no formal isobolographic or combination-index analysis was performed.

### 2.5. Disease-State Dependence

Comparisons between identical monotherapies in healthy and diabetic animals indicated that all drugs were highly diabetic disease state-dependent (Table 5). The biggest difference was with amlodipine (5.34% of healthy participants compared to 8.47% of diabetes participants; difference 3.13 points, *p* = 1.7 × 10^−4^), followed by glimepiride (2.40 points) and bisoprolol (2.33 points). Therefore, it is important to recognize that the effects observed in normoglycemic individuals are not necessarily the same in diabetes, and to evaluate them in a disease-specific manner when choosing combination regimens.

### 2.6. Machine Learning and Unsupervised Validation

**Figure 4:**
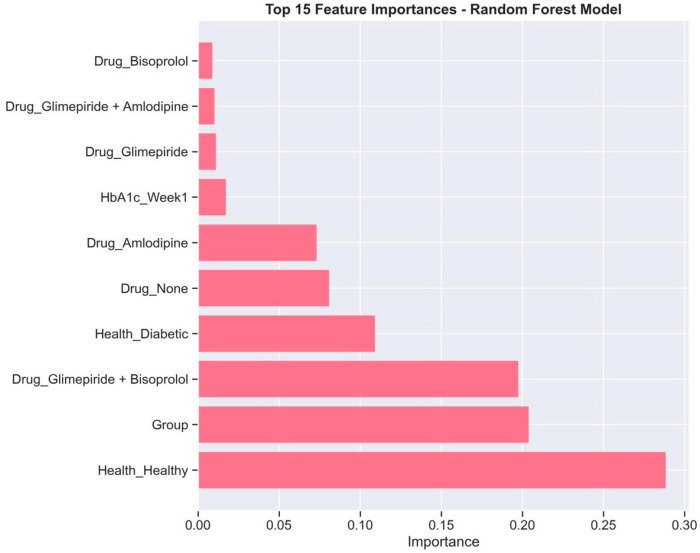
Random Forest feature importances (Gini) for week-11 HbA1c prediction. Health status and treatment group assignment dominate; baseline HbA1c is a minor contributor.

**Figure 5:**
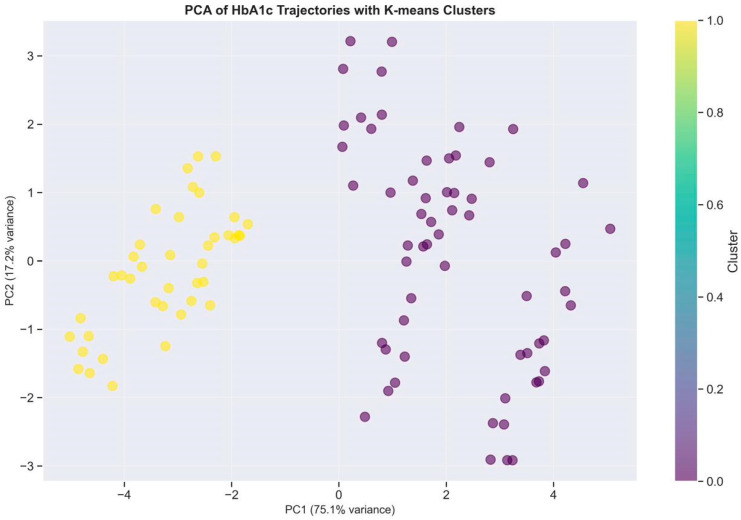
Longitudinal HbA1c data projected to PCA and colored by K-means cluster (k = 2). Effective regimens (left cluster) are clearly distinguished from deteriorating regimens (right cluster), thereby providing model-free support for the statistical results.

**Figure 6:**
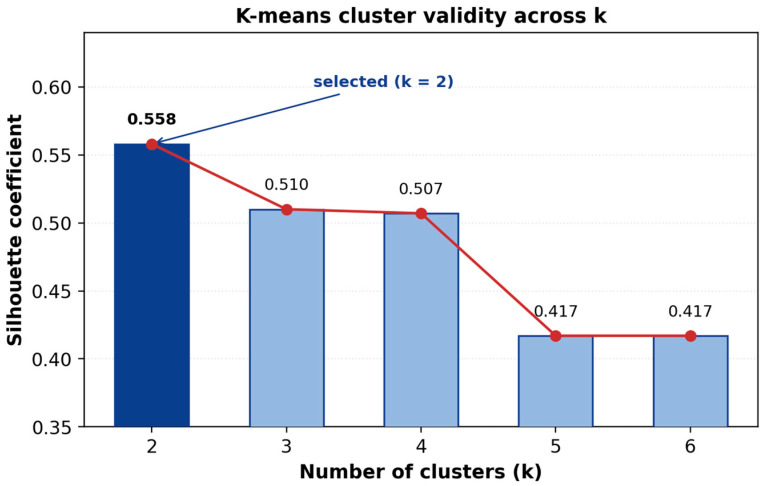
K-means cluster validity across candidate cluster numbers. The silhouette coefficient is maximal at k = 2 (0.558) and decreases for k = 3–6, supporting the two-cluster solution shown in Figure 5. Corresponding values are tabulated in Supplementary Table S9.

A Random-Forest regression model predicting week-11 HbA1c from baseline HbA1c, treatment assignment and health status achieved excellent accuracy (R^2^ = 0.985, RMSE = 0.207, MAE = 0.163). Feature importance analysis (Figure 4) showed that health status (28.8%) and treatment group identity (20.4%) were the most important features, while baseline HbA1c was the least important, accounting for only 9.2%. This implies that the nature of the treatment received and the underlying health status have a greater determining influence on the outcome than the baseline HbA1C level. The three components obtained from the principal component analysis explained 98.4% of the variance, and K-means clustering (k = 2, silhouette = 0.558) automatically classified the effective regimen (glimepiride + bisoprolol) as distinct from both the healthy and deteriorating groups (Figure 5). This unsupervised structure, determined without group labels, confirms the previously obtained statistical treatment hierarchy. To verify the choice of cluster number, the silhouette coefficient was evaluated across k = 2–6; it peaked at k = 2 (0.558) and declined for larger values (k = 3, 0.510; k = 4, 0.507; k = 5, 0.417; k = 6, 0.417; Figure 6 and Supplementary Table S9), confirming that a two-cluster partition best represented the data. Pharmacologically, the first cluster grouped the effective, glycemia-stabilizing profiles—glimepiride+bisoprolol together with all healthy groups—characterized by low and stable HbA1c, whereas the second grouped the deteriorating profiles—the untreated diabetic control, amlodipine monotherapy and the remaining diabetic monotherapies—characterized by progressively rising HbA1c; glimepiride+amlodipine occupied an intermediate position within the effective-leaning cluster, consistent with its glucose-neutral additive behavior.

## 3. Discussion

The present controlled preclinical study aimed to answer an important but underinvestigated clinical question: does the type of antihypertensive drug affect the glycemic effect of sulfonylurea? Three findings stand out. First, co-administration of either bisoprolol or amlodipine with glimepiride did not impair glycemic control; on the contrary, the bisoprolol combination produced the best control of any diabetic regimen, achieving HbA1c levels that were statistically indistinguishable from those in healthy animals. Second, the benefit of adding bisoprolol exceeded the additive effect expected from the two drugs individually, indicating a synergistic interaction, whereas amlodipine contributed only a modest additive effect. Third, every agent’s effect was strongly disease-state dependent, so observations in healthy animals could not predict diabetic outcomes.

The superior performance of glimepiride+bisoprolol is biologically plausible. Excessive sympathetic activation is a recognized feature of T2DM and contributes to insulin resistance and impaired β-cell function; cardioselective β1-blockade may alleviate part of this catecholaminergic burden without the unfavorable metabolic effects historically attributed to non-selective β-blockers [6,7]. A complementary mechanism—β-blocker—mediated reduction in sympathetically driven hepatic glucose output alongside glimepiride-stimulated insulin secretion—could account for the greater-than-additive HbA1c reduction observed here. These interpretations remain hypotheses, since the present design did not include insulin, lipid, blood pressure or molecular endpoints.

Amlodipine monotherapy did not attenuate the progressive rise in HbA1c, and endpoint values approached those of untreated diabetic controls. Rather than indicating active metabolic harm, this pattern is consistent with the well-documented absence of intrinsic glucose-lowering activity in dihydropyridine CCBs [8]: amlodipine controls blood pressure but does not itself improve glycemia, so when given without an antihyperglycemic agent, the natural disease trajectory continues largely unopposed. Importantly, when amlodipine was combined with glimepiride, glycemic control was preserved and modestly improved, reaffirming its glucose-neutral profile and suitability as an antihypertensive in patients already receiving effective antihyperglycemic therapy, including in fixed-dose antihypertensive combinations frequently used to manage comorbid hypertension [22].

Convergence across independent analytical strategies strengthens these conclusions. Non-parametric hypothesis testing, a high-accuracy Random Forest model, and unsupervised clustering each placed glimepiride+bisoprolol, with the healthy phenotype and isolated amlodipine monotherapy and the untreated control as the poorest performers. The importance of treatment and disease-state features in the model, rather than baseline HbA1c, reflects the fundamental tenet of clinical care that suboptimal glycemia at presentation can be overcome with the appropriate choice of medications.

The findings confirm the initial conclusion of the experiments, suggest that co-administration of antihypertensive drugs does not impair the glucose-lowering effect of glimepiride, and establish a definite benefit of cardioselective β-blockade. They also reflect clinical experience, which has shown that metabolically modern β-blockers are better tolerated in diabetic patients than older non-selective agents [6,7].

Several caveats define the boundaries within which the present conclusions should be interpreted. First, the evaluation relied on a single primary endpoint, glycated hemoglobin (HbA1c); insulin, lipid, blood pressure and histological parameters were not measured, so the mechanistic interpretations offered above—particularly the proposed sympatholytic and hepatic glucose output contributions of bisoprolol—remain hypotheses rather than demonstrated mechanisms. Second, body weight was recorded only at weeks 1, 6 and 11; this sparse sampling limits the resolution with which drug effects on metabolic and weight trajectories can be described, and more frequent weighing in future work, analyzed jointly with the HbA1c trend, would better characterize the dynamic metabolic state. Third, HbA1c was quantified by a single point-of-care boronate-affinity immunoassay without a parallel orthogonal method (for example, HPLC or capillary electrophoresis), so the analytical robustness of the measurement was not independently cross-validated. Fourth, hepatic and renal safety were not assessed; biomarkers of liver and kidney function, together with histopathology, are needed to establish the safety of long-term combination therapy. Fifth, the streptozotocin/high-fat model does not fully reproduce the pathophysiology of human T2DM; only male Wistar rats were studied (precluding analysis of sex differences), and doses were fixed and non-titrated (precluding dose–response assessment). Finally, repeated-measures data were analyzed with paired non-parametric tests rather than a mixed-effects model, and the synergy described is operational rather than formally demonstrated. Accordingly, the conclusions apply to this specific experimental model and endpoint. They should be extrapolated to other species, females, titrated dosing and clinical populations only with appropriate confirmatory studies. From a formulation perspective, advanced oral delivery platforms—such as mesoporous silica carriers developed to enhance the dissolution and bioavailability of poorly soluble cardiovascular drugs—represent a complementary avenue for optimizing these agents [23].

## 4. Materials and Methods

### 4.1. Animals and Ethics

The adult male Wistar rats (250–300 g, 8–10 weeks old) were kept under standard laboratory conditions (22 ± 2 °C, 12 h light/dark cycle) with free access to water. All experimental procedures were approved by the Animal Ethics Committee of Al-Ahliyya Amman University (reference 8/2018 EC-AAC; review date 27 August 2018) and carried out in accordance with the relevant institutional guidelines and regulations. The study has been reported in accordance with the ARRIVE guidelines 2.0 [24,25].

### 4.2. Experimental Groups

Animals were allocated to ten parallel groups (*n* = 10 each): (1) healthy control, (2) diabetic control, (3) diabetic + glimepiride, (4) healthy + glimepiride, (5) diabetic + glimepiride + bisoprolol, (6) diabetic + glimepiride + amlodipine, (7) healthy + bisoprolol, (8) healthy + amlodipine, (9) diabetic + bisoprolol, and (10) diabetic + amlodipine. Disease-state specificity was assessed in the healthy and diabetic monotherapy arms, and additive or synergistic modulation was assessed in the two diabetic combination arms.

### 4.3. Induction of Type 2 Diabetes

Insulin resistance was induced by four weeks on a validated high-fat diet (≈60% of calories from fat), followed by a single low-dose STZ in citrate buffer (35 mg/kg) after 16 h of fasting [15]. Sustained fasting blood glucose level ≥ 200 mg/dL at 72 h after injection (Accu-Chek Performa, Roche, Basel, Switzerland) was used for confirmation of diabetes; only animals that were confirmed were included in the diabetic arms. Healthy controls were fed with regular chow and given vehicle injections.

### 4.4. Drug Administration

The doses were determined using physiologically relevant human-equivalent dose scaling from standard clinical doses of glimepiride (0.5 mg/kg), bisoprolol (10 mg/kg), and amlodipine (5 mg/kg), and treatment commenced as soon as diabetes was confirmed.

### 4.5. Outcome Measurements

The primary outcome, HbA1c, was measured weekly from week 1 to week 11, using a species-specific i-CHROMA boronate-affinity fluorescence-immunoassay point-of-care analyzer (Boditech Med Inc., Chuncheon, Republic of Korea) with species-specific cartridges (100 rats × 11 weeks = 1100 measurements) [19,20]. Secondary outcome measures included fasting blood glucose (weeks 1–3) and body weight (weeks 1, 6 and 11).

### 4.6. Statistical Analysis

Distributional assumptions were assessed with the Shapiro–Wilk test (normality) and Levene’s test (variance homogeneity). Because homogeneity was rejected, non-parametric methods were mainly used: the Kruskal–Wallis H-test for global comparison, Dunn’s test with Bonferroni correction for pairwise contrasts (45 comparisons), Mann–Whitney U for healthy-versus-diabetic contrasts, and the Wilcoxon signed-rank test for paired week 1-versus-week 11 changes. Effect sizes (mean difference, percentage change and Cohen’s d) were computed where appropriate. Statistical significance was set at α = 0.05.

### 4.7. Machine Learning and Clustering

A Random Forest regression model [21] (500 trees, maximum depth 20, minimum samples to split 5; 75/25 train/test split) predicted week-11 HbA1c from baseline HbA1c and one-hot-encoded treatment and health status variables; feature importances were derived from mean decrease in impurity. Principal component analysis was applied to standardized weekly HbA1c profiles, and K-means clustering (k evaluated from 2 to 6) was assessed using the silhouette coefficient. All analyses were performed in Python 3.10 using pandas, scipy, statsmodels, scikit-learn [22] and scikit-posthocs.

## 5. Conclusions

In this 11-week streptozotocin/high-fat T2DM rat model, co-administration of the cardioselective β-blocker bisoprolol or the dihydropyridine CCB amlodipine did not impair the glycemic efficacy of glimepiride. Bisoprolol enhanced glimepiride’s effect—uniquely producing a declining HbA1c trajectory and near-normal endpoint control with an apparent synergistic interaction—whereas amlodipine preserved control consistent with a glucose-neutral profile. All agents exhibited strong disease-state dependence, and three independent analytical approaches converged on the same treatment hierarchy. Pending clinical confirmation, these findings support cardioselective β-blockade as a metabolically favorable antihypertensive partner for sulfonylurea therapy in patients with coexisting hypertension and T2DM. More broadly, these findings offer a preclinical, evidence-based rationale for prioritizing metabolically favorable antihypertensive agents in patients who require concurrent glucose- and blood pressure-lowering therapy, with the potential to improve glycemic outcomes, simplify combination regimens, and reduce the risk of adverse drug–disease interactions. Future studies should extend this work with a broader biochemical and cardiometabolic panel (insulin, lipids and blood pressure), hepatic and renal safety monitoring, denser longitudinal sampling, both sexes, and titrated dosing. They should ultimately progress to controlled clinical trials to confirm the cardiometabolic benefit of combining cardioselective β-blockade with sulfonylurea therapy.

## Figures and Tables

**Table 1:** Body weight (mean ± SD, g) at weeks 1, 6 and 11. *n* = 10 per group. Untreated diabetic animals showed progressive weight loss; glimepiride-containing regimens attenuated this catabolic response.

Group	Week 1 (g)	Week 6 (g)	Week 11 (g)
Healthy control	234.8 ± 22.0	237.9 ± 22.8	242.3 ± 21.1
Healthy + glimepiride	237.5 ± 16.4	245.8 ± 16.8	229.2 ± 17.2
Healthy + bisoprolol	241.9 ± 17.3	247.8 ± 16.7	256.1 ± 15.9
Healthy + amlodipine	242.4 ± 14.8	251.1 ± 15.3	261.0 ± 15.8
Diabetic control	260.4 ± 11.6	211.6 ± 9.2	103.9 ± 5.7
Diabetic + glimepiride	247.6 ± 14.7	228.0 ± 11.8	200.3 ± 7.4
Diabetic + glim. + bisoprolol	261.9 ± 12.4	232.5 ± 9.9	202.1 ± 6.0
Diabetic + glim. + amlodipine	258.6 ± 12.4	229.9 ± 9.9	195.0 ± 6.4
Diabetic + bisoprolol	263.2 ± 11.6	236.8 ± 9.2	198.8 ± 32.1
Diabetic + amlodipine	262.1 ± 11.6	233.3 ± 11.6	192.7 ± 32.1

**Table 2:** Week-11 HbA1c descriptive statistics. *n* = 10 per group. Groups are ordered by mean within disease states.

Group	Mean (%)	SD	Median (%)	Range (%)
Diabetic + glim. + bisoprolol	4.37	0.15	4.40	4.1–4.6
Diabetic + glim. + amlodipine	5.99	0.12	6.00	5.8–6.2
Diabetic + glimepiride	6.44	0.27	6.45	6.1–6.9
Diabetic + bisoprolol	6.61	0.10	6.60	6.5–6.8
Diabetic + amlodipine	8.47	0.20	8.45	8.2–8.8
Diabetic control	9.31	0.18	9.30	9.1–9.6
Healthy + glimepiride	4.04	0.31	4.05	3.6–4.6
Healthy + bisoprolol	4.28	0.29	4.15	4.0–4.7
Healthy control	5.00	0.42	5.05	4.3–5.5
Healthy + amlodipine	5.34	0.25	5.40	4.7–5.6

**Table 3:** Selected Dunn’s post hoc comparisons (Bonferroni-adjusted). *** *p* < 0.001; ns, not significant. The complete 10 × 10 comparison matrix is provided in Supplementary Table S1.

Comparison	Δ HbA1c (Points)	Significance
Diabetic control vs. glim+bisoprolol	4.94	***
Diabetic control vs. glim+amlodipine	3.32	***
Diabetic control vs. glimepiride	2.87	***
Diabetic control vs. bisoprolol	2.70	***
Diabetic control vs. amlodipine	0.84	ns
Glimepiride vs. glim+bisoprolol	2.07	***
Glimepiride vs. glim+amlodipine	0.45	***
Amlodipine vs. glim+bisoprolol	4.10	***

**Table 4:** Longitudinal HbA1c change (week 1 → week 11) in diabetic groups (Wilcoxon signed-rank). Only glimepiride + bisoprolol improved; all other regimens deteriorated.

Group	Baseline (%)	Week 11 (%)	Δ (Points)	*p*
Diabetic + glim. + bisoprolol	5.03	4.37	−0.66	0.004
Diabetic + glim. + amlodipine	4.65	5.99	+1.34	0.002
Diabetic + glimepiride	4.90	6.44	+1.54	0.002
Diabetic + bisoprolol	4.30	6.61	+2.31	0.002
Diabetic + amlodipine	4.16	8.47	+4.31	0.002
Diabetic control	4.81	9.31	+4.50	0.002

**Table 5:** Disease-state specificity of monotherapy responses (week-11 HbA1c; Mann–Whitney U). Difference = diabetic − healthy. *** *p* < 0.001.

Drug	Healthy (%)	Diabetic (%)	Difference (Points)
Glimepiride	4.04	6.44	2.40 ***
Bisoprolol	4.28	6.61	2.33 ***
Amlodipine	5.34	8.47	3.13 ***

## Data Availability

The original contributions presented in this study are included in the article and Supplementary Material. Further inquiries can be directed to the corresponding author.

## Supplementary Materials

The following supporting information can be downloaded at https://www.mdpi.com/article/10.3390/ph19071064/s1. Table S1. Complete pairwise comparison matrix (Dunn’s test, Bonferroni-adjusted); Table S2. Group-wise Shapiro–Wilk normality results; Table S3. Experimental group design; Table S4. Dosing regimen and human-equivalent-dose basis; Table S5. Week-11 HbA1c descriptive summary by group; Table S6. Longitudinal HbA1c change (week 1 → week 11), diabetic groups; Table S7. Disease-state specificity of monotherapy responses; Table S8. Random-Forest configuration and performance; Table S9. PCA / K-means clustering summary and software environment; Table S10. Individual-level endpoint variability by group; Figure S1. HbA1c summary heatmap (all ten groups); Figure S2. Data-processing and analysis pipeline.

## References

[1] Cheung B.M., Li C. (2012). Diabetes and hypertension: Is there a common metabolic pathway?. Curr. Atheroscler. Rep..

[2] Petrie J.R., Guzik T.J., Touyz R.M. (2018). Diabetes, hypertension, and cardiovascular disease: Clinical insights and vascular mechanisms. Can. J. Cardiol..

[3] American Diabetes Association (2014). Diagnosis and classification of diabetes mellitus. Diabetes Care.

[4] Hammad A.M., Al-Qerem W., Alasmari F., Ling J., Qarqaz R., Alaqabani H. (2022). Identifying Drug-Therapy Problems among Syrian Refugees in Zaatari Refugee Camp. Int. J. Environ. Res. Public Health.

[5] American Diabetes Association Professional Practice Committee (2024). 2. Diagnosis and Classification of Diabetes: Standards of Care in Diabetes—2024. Diabetes Care.

[6] Bell D.S. (1999). Beta-adrenergic blocking agents in patients with diabetes--friend and foe. Endocr. Pract..

[7] Bakris G.L., Fonseca V., Katholi R.E., McGill J.B., Messerli F.H., Phillips R.A., Raskin P., Wright J.T., Oakes R., Lukas M.A. (2004). Metabolic effects of carvedilol vs metoprolol in patients with type 2 diabetes mellitus and hypertension. JAMA.

[8] Mason R.P., Marche P., Hintze T.H. (2003). Novel vascular biology of third-generation calcium-channel antagonists. Arterioscler. Thromb. Vasc. Biol..

[9] Davis S.N. (2004). The role of glimepiride in the effective management of type 2 diabetes. J. Diabetes Complicat..

[10] Hamad M., Abu Dayyih W., Rafal A., Abu Dayyih A., Al Ani I., Mallah E., Salih H., Zakarya Z., Arafat T. (2017). The effect of some fruit juices on the pharmacokinetics of glimepiride in rat plasma by using HPLC-MS. Biomed. Pharmacol. J..

[11] Ahmad R.A., Hailat M.M., Jaber M.A., Alkhawaja B.A., Rasras A.A., Al-Shdefat R., Mallah E., Abu Dayyih W. (2021). Development and validation of an RP-HPLC method for the simultaneous determination of empagliflozin, pioglitazone and metformin in pharmaceutical dosage form. Acta Pol. Pharm..

[12] Alkather Z., Hailat M., Al-Shdefat R., Abu Dayyih W. (2021). Development and validation of a new HPLC method for the simultaneous determination of five gliptins. Curr. Pharm. Anal..

[13] Deeks E.D., Keating G.M. (2007). Bisoprolol: A review of its use in chronic heart failure. Drugs.

[14] Furman B.L. (2015). Streptozotocin-induced diabetic models in mice and rats. Curr. Protoc. Pharmacol..

[15] Srinivasan K., Viswanad B., Asrat L., Kaul C.L., Ramarao P. (2005). Combination of high-fat diet-fed and low-dose streptozotocin-treated rat: A model for type 2 diabetes and pharmacological screening. Pharmacol. Res..

[16] Zakaraya Z.Z., AlTamimi L., Hailat M., Ahmad M.N., Qinna N.A., Ghanim B.Y., Saadh M.J., Al-Dmour N., Dayyih W.A. (2022). Ameliorative effect of selenium yeast in combination with pioglitazone on diabetes outcomes in streptozotocin-induced. J. Popul. Ther. Clin. Pharmacol..

[17] Tamimi L.N., Zakaraya Z., Hailat M., Abu Dayyih W., Daoud E., Abed A., Saadh M.J., Majeed B., Abumansour H., Aburumman A. (2023). Anti-diabetic effect of cotreatment with resveratrol and pioglitazone in diabetic rats. Eur. Rev. Med. Pharmacol. Sci..

[18] Al Tamimi L., Zakaraya Z.Z., Hailat M., Ahmad M.N., Qinna N.A., Hamad M.F., Abu Dayyih W. (2024). The relationship between insulin resistance and glycosylated haemoglobin in streptozotocin-induced diabetic rats. J. Adv. Pharm. Technol. Res..

[19] Sherwani S.I., Khan H.A., Ekhzaimy A., Masood A., Sakharkar M.K. (2016). Significance of HbA1c test in diagnosis and prognosis of diabetic patients. Biomark. Insights.

[20] Breiman L. (2001). Random forests. Mach. Learn..

[21] Pedregosa F., Varoquaux G., Gramfort A., Michel V., Thirion B., Grisel O., Blondel M., Prettenhofer P., Weiss R., Dubourg V. (2011). Scikit-learn: Machine learning in Python. J. Mach. Learn. Res..

[22] Said R., Arafat B., Arafat T., Mallah E. (2021). An LC-MS/MS method for determination of triple drugs combination of valsartan, amlodipine and hydrochlorothiazide in human plasma for bioequivalence study. Curr. Pharm. Anal..

[23] Mahdi H., Muhana F., Al-Ani I., Al-Sanabrah A. (2022). Preparation and evaluation of SBA-16, ZSM-5 and MCM-41 mesoporous silica nanoparticles as drug delivery system for carvedilol. Int. J. Drug Deliv. Technol..

[24] Percie du Sert N., Hurst V., Ahluwalia A., Alam S., Avey M.T., Baker M., Browne W.J., Clark A., Cuthill I.C., Dirnagl U. (2020). The ARRIVE guidelines 2.0: Updated guidelines for reporting animal research. PLoS Biol..

[25] Sarkar A.K., Ghosh D., Das A., Selvan P.S., Gowda K.V., Mandal U., Bose A., Agarwal S., Bhaumik U., Pal T.K. (2008). Simultaneous determination of metoprolol succinate and amlodipine besylate in human plasma by LC–MS/MS and its application in a bioequivalence study. J. Chromatogr. B.

