# Supplementary material for: Bisoprolol and Amlodipine Co-Administration with Glimepiride in a Diabetic Rat Model: A Statistical and Machine Learning Analysis
Source: Pharmaceuticals (Basel). 2026 Jul 10;19(7):1064. doi: 10.3390/ph19071064 (PMC13415167; doi:10.3390/ph19071064)
Supplement: Supplementary file 1 [file pharmaceuticals-19-01064-s001.zip › pharmaceuticals-4392816-supplementary.pdf]

Supplementary Materials

Differential Effects of Bisoprolol and Amlodipine Co-administration on Glimepiride-Mediated Glycaemic Control in a Streptozotocin-Induced Type 2 Diabetes Mellitus Rat Model: An Integrated Statistical and Machine-Learning Analysis

Contents

Table S1. Complete pairwise comparison matrix (Dunn's test, Bonferroni-adjusted).
Table S2. Group-wise Shapiro–Wilk normality results.
Table S3. Experimental group design.
Table S4. Dosing regimen and human-equivalent-dose basis.
Table S5. Week-11 HbA1c descriptive summary by group.
Table S6. Longitudinal HbA1c change (week 1 → week 11), diabetic groups.
Table S7. Disease-state specificity of monotherapy responses.
Table S8. Random-Forest configuration and performance.
Table S9. PCA / K-means clustering summary and software environment.
Table S10. Individual-level endpoint variability by group.
Figure S1. HbA1c summary heatmap (all ten groups).
Figure S2. Data-processing and analysis pipeline.

Table S1. Pairwise comparison matrix (Dunn's test, Bonferroni-adjusted p-values)

Group	1 HC	2 DC	3 D+G	4 H+G	5 D+G+B	6 D+G+A	7 H+B	8 H+A	9 D+B	10 D+A
**1 HC**	—	**<0.001**	0.606	1.00	1.00	1.00	1.00	1.00	0.183	**0.005**
**2 DC**	**<0.001**	—	1.00	**<0.001**	**<0.001**	0.102	**<0.001**	**0.002**	1.00	1.00
**3 D+G**	0.606	1.00	—	**<0.001**	**0.015**	1.00	**0.005**	1.00	1.00	1.00
**4 H+G**	1.00	**<0.001**	**<0.001**	—	1.00	**0.020**	1.00	0.526	**<0.001**	**<0.001**
**5 D+G+B**	1.00	**<0.001**	**0.015**	1.00	—	0.328	1.00	1.00	**0.003**	**<0.001**
**6 D+G+A**	1.00	0.102	1.00	**0.020**	0.328	—	0.130	1.00	1.00	1.00
**7 H+B**	1.00	**<0.001**	**0.005**	1.00	1.00	0.130	—	1.00	**<0.001**	**<0.001**
**8 H+A**	1.00	**0.002**	1.00	0.526	1.00	1.00	1.00	—	0.970	**0.048**
**9 D+B**	0.183	1.00	1.00	**<0.001**	**0.003**	1.00	**<0.001**	0.970	—	1.00
**10 D+A**	**0.005**	1.00	1.00	**<0.001**	**<0.001**	1.00	**<0.001**	**0.048**	1.00	—

Table S1. Complete 10 × 10 pairwise comparison matrix (Dunn's test, Bonferroni-adjusted p-values) for week-11 HbA1c. Group keys: HC, healthy control; DC, untreated diabetic control; D+G, diabetic+glimepiride; H+G, healthy+glimepiride; D+G+B, diabetic+glimepiride+bisoprolol; D+G+A, diabetic+glimepiride+amlodipine; H+B, healthy+bisoprolol; H+A, healthy+amlodipine; D+B, diabetic+bisoprolol; D+A, diabetic+amlodipine. The 45 unique comparisons used a Bonferroni-adjusted threshold (0.05/45 ≈ 0.0011); shaded/bold cells denote $p < 0.05$. Diabetic+glimepiride+bisoprolol (D+G+B) differs from every diabetic group ($p \leq 0.015$) yet is equivalent to all healthy groups ($p \geq 0.328$); diabetic+amlodipine (D+A) does not differ from the untreated diabetic control ($p = 1.00$). The diagonal is not applicable (—).

Table S2. Group-wise Shapiro–Wilk normality results

Group	n	W	p	Normal (α = 0.05)
Healthy control	10	0.9153	0.3190	Yes
Untreated diabetic control	10	0.9047	0.2463	Yes
Diabetic + glimepiride	10	0.9402	0.5548	Yes

Group	n	W	p	Normal (α = 0.05)
Healthy + glimepiride	10	0.9566	0.7471	Yes
Diabetic + glimepiride + bisoprolol	10	0.9662	0.8540	Yes
Diabetic + glimepiride + amlodipine	10	0.9519	0.6915	Yes
Healthy + bisoprolol	10	0.8282	0.0318	**No**
Healthy + amlodipine	10	0.7975	0.0135	**No**
Diabetic + bisoprolol	10	0.8858	0.1520	Yes
Diabetic + amlodipine	10	0.9085	0.2711	Yes

Table S2. Group-wise Shapiro–Wilk normality test results for week-11 HbA1c (decisions at α = 0.05). Eight of the ten groups were consistent with normality; healthy + bisoprolol and healthy + amlodipine deviated ($p < 0.05$). Levene's test rejected homogeneity of variance ($L = 2.9350$, $p = 0.004247$), which motivated the non-parametric analytical framework.

Table S3. Experimental group design

Group	Health state	Treatment	n
1	Healthy	Untreated control (vehicle)	10
2	Diabetic	Untreated control (vehicle)	10
3	Diabetic	Glimepiride	10
4	Healthy	Glimepiride	10
5	Diabetic	Glimepiride + bisoprolol	10
6	Diabetic	Glimepiride + amlodipine	10
7	Healthy	Bisoprolol	10
8	Healthy	Amlodipine	10
9	Diabetic	Bisoprolol	10
10	Diabetic	Amlodipine	10

Table S3. Allocation of the 100 male Wistar rats to ten parallel groups (n = 10 each). Disease-state specificity was assessed across matched healthy and diabetic monotherapy arms; additive/synergistic modulation was assessed in the two diabetic combination arms.

Table S4. Dosing regimen and human-equivalent-dose basis

Drug	Dose (mg/kg)	Route	Notes
Glimepiride	0.5	Oral	Sulfonylurea; HED-scaled (human ≈2–4 mg/day)
Bisoprolol	10	Oral	β1-selective β-blocker; HED-scaled (human ≈5–10 mg/day)
Amlodipine	5	Oral	Dihydropyridine CCB; HED-scaled (human ≈5–10 mg/day)
Streptozotocin	35	i.p.	Single low dose in citrate buffer (pH 4.5), after fast

Table S4. Doses were derived by physiologically relevant human-equivalent-dose (HED) scaling from standard clinical doses. Treatment commenced once diabetes was confirmed (fasting blood glucose ≥ 200 mg/dL at 72 h post-injection). Type 2 diabetes was induced by high-fat diet (≈60% of calories from fat) followed by low-dose streptozotocin.

Table S5. Week-11 HbA1c descriptive summary

Group	Mean ± SD (%)	Median (%)	Min	Max
Glimepiride + bisoprolol (D)	4.37 ± 0.15	4.40	4.1	4.6
Healthy + glimepiride	4.04 ± 0.31	4.05	3.6	4.6
Healthy + bisoprolol	4.28 ± 0.29	4.15	4.0	4.7
Healthy control	5.00 ± 0.42	5.05	4.3	5.5
Healthy + amlodipine	5.34 ± 0.25	5.40	4.7	5.6
Glimepiride + amlodipine (D)	5.99 ± 0.12	6.00	5.8	6.2
Glimepiride (D)	6.44 ± 0.27	6.45	6.1	6.9
Bisoprolol (D)	6.61 ± 0.10	6.60	6.5	6.8
Amlodipine (D)	8.47 ± 0.20	8.45	8.2	8.8
Untreated diabetic control	9.31 ± 0.18	9.30	9.1	9.6

Table S5. Week-11 HbA1c by group (n = 10 each), ordered to show healthy (green) and diabetic tiers. Endpoint HbA1c spanned 4.94 percentage points (4.37–9.31% across diabetic groups). Within-group standard deviations were low (0.10–0.42) and medians approximated means, indicating consistent within-group responses.

Table S6. Longitudinal HbA1c change (week 1 → week 11), diabetic groups

Diabetic group	Baseline (%)	Week 11 (%)	Δ (pp)	Direction	Wilcoxon p
**Glimepiride + bisoprolol**	**5.03**	**4.37**	**−0.66**	**Improved**	**0.003906**
Glimepiride + amlodipine	4.65	5.99	+1.34	Deteriorated	0.001953
Glimepiride	4.90	6.44	+1.54	Deteriorated	0.001953
Bisoprolol	4.30	6.61	+2.31	Deteriorated	0.001953
Amlodipine	4.16	8.47	+4.31	Deteriorated	0.001953
Untreated diabetic control	4.81	9.31	+4.50	Deteriorated	0.001953

Table S6. Within-group change in HbA1c from week 1 to week 11 (Wilcoxon signed-rank). Only glimepiride + bisoprolol showed a declining trajectory; all other diabetic regimens rose. pp, percentage points.

Table S7. Disease-state specificity of monotherapy responses (Mann–Whitney U)

Monotherapy	Healthy HbA1c (%)	Diabetic HbA1c (%)	Difference (pp)	p
Glimepiride	4.04	6.44	2.40	0.000177
Bisoprolol	4.28	6.61	2.33	0.000162
Amlodipine	5.34	8.47	3.13	0.000165

Table S7. Difference between matched diabetic and healthy monotherapy arms (difference = diabetic − healthy). All monotherapies were strongly disease-state dependent (all $p < 0.0002$); amlodipine showed the largest divergence. pp, percentage points.

Table S8. Random-Forest configuration and performance

Parameter / metric	Value
Model	Random-Forest regression
Number of trees	500
Maximum depth	20
Minimum samples to split	5

Parameter / metric	Value
Train / test split	75 / 25 (75 train, 25 test)
Predictors	Baseline HbA1c; one-hot treatment; health status
Importance metric	Mean decrease in impurity (Gini)
R^2 (test)	0.9851
MSE	0.0428
RMSE	0.207
MAE	0.163
Feature importance — health status	28.8%
Feature importance — treatment-group identity	20.4%
Feature importance — baseline HbA1c	9.2%

Table S8. Random-Forest regression specification and performance for week-11 HbA1c prediction. Health status and treatment-group assignment were the dominant predictors; baseline HbA1c contributed least, indicating that disease state and treatment govern the outcome more than initial glycaemia.

Table S9. PCA / K-means clustering summary and software environment

Principal component	Variance explained (%)	Cumulative (%)
PC1	75.1	75.1
PC2	17.2	92.3
PC3	6.1	98.4

K-means k	Silhouette coefficient
**2**	**0.558 (selected)**
3	0.510
4	0.507
5	0.417
6	0.417

Table S9. Unsupervised analysis: PCA variance decomposition (top) and K-means silhouette scores across k (bottom). PCA was applied to standardised weekly HbA1c profiles; three components captured 98.4% of variance. K-means maximised the silhouette coefficient at k = 2, separating effective regimens and healthy groups from deteriorating regimens. Analyses were performed in Python 3.10 (pandas, scipy, statsmodels, scikit-learn, scikit-posthocs); significance threshold α = 0.05.

Table S10. Individual-level endpoint variability by group

Group	n	Mean Δ (pp)	SD of Δ	Range of Δ (pp)
Diabetic + glimepiride + bisoprolol	10	−0.66	0.38	−1.20 to +0.10
Healthy + glimepiride	10	−0.77	0.25	−1.10 to −0.30
Healthy + bisoprolol	10	−0.41	0.27	−0.80 to 0.00

Group	n	Mean Δ (pp)	SD of Δ	Range of Δ (pp)
Healthy control	10	+0.25	0.08	+0.20 to +0.40
Healthy + amlodipine	10	+0.59	0.18	+0.40 to +0.80
Diabetic + glimepiride + amlodipine	10	+1.34	0.28	+0.90 to +1.70
Diabetic + glimepiride	10	+1.54	0.27	+1.10 to +2.00
Diabetic + bisoprolol	10	+2.31	0.32	+1.80 to +2.70
Diabetic + amlodipine	10	+4.31	0.30	+3.90 to +4.80
Untreated diabetic control	10	+4.50	0.41	+3.80 to +5.30

Table S10. Individual-level change in HbA1c (week 1 → week 11): group mean, within-group SD and observed range (n = 100 rats). Directionality was 100% consistent within groups (every glimepiride + bisoprolol animal improved or stabilised; every untreated diabetic-control animal deteriorated), and the narrow SDs indicate reproducible pharmacodynamic responses rather than responder/non-responder averaging. pp, percentage points.

Figure S1. HbA1c summary heatmap

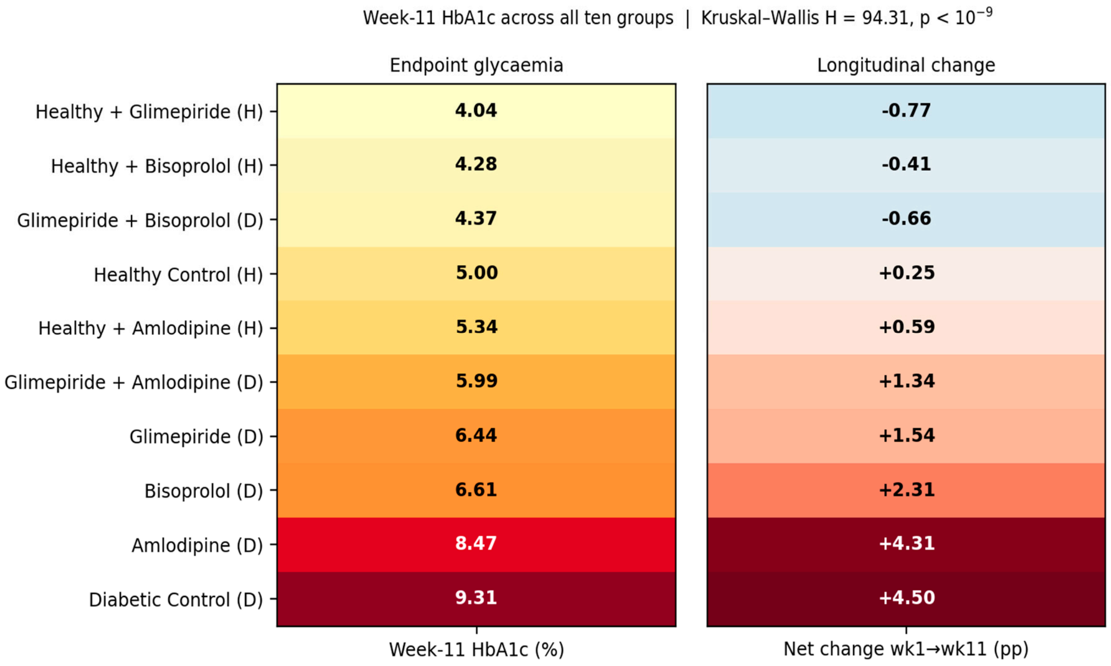

Figure S1. Summary heatmap of week-11 (endpoint) HbA1c and net week 1 → week 11 change for all ten groups. Groups are ordered by ascending endpoint HbA1c; (H) healthy, (D) diabetic. Left panel colour scales with endpoint HbA1c; right panel colour scales with the signed longitudinal change (blue = improvement, red = deterioration). Only the diabetic glimepiride + bisoprolol regimen (and the glimepiride/bisoprolol healthy arms) shows a declining trajectory; amlodipine monotherapy and the untreated diabetic control rise most steeply.

Figure S2. Data-processing and analysis pipeline

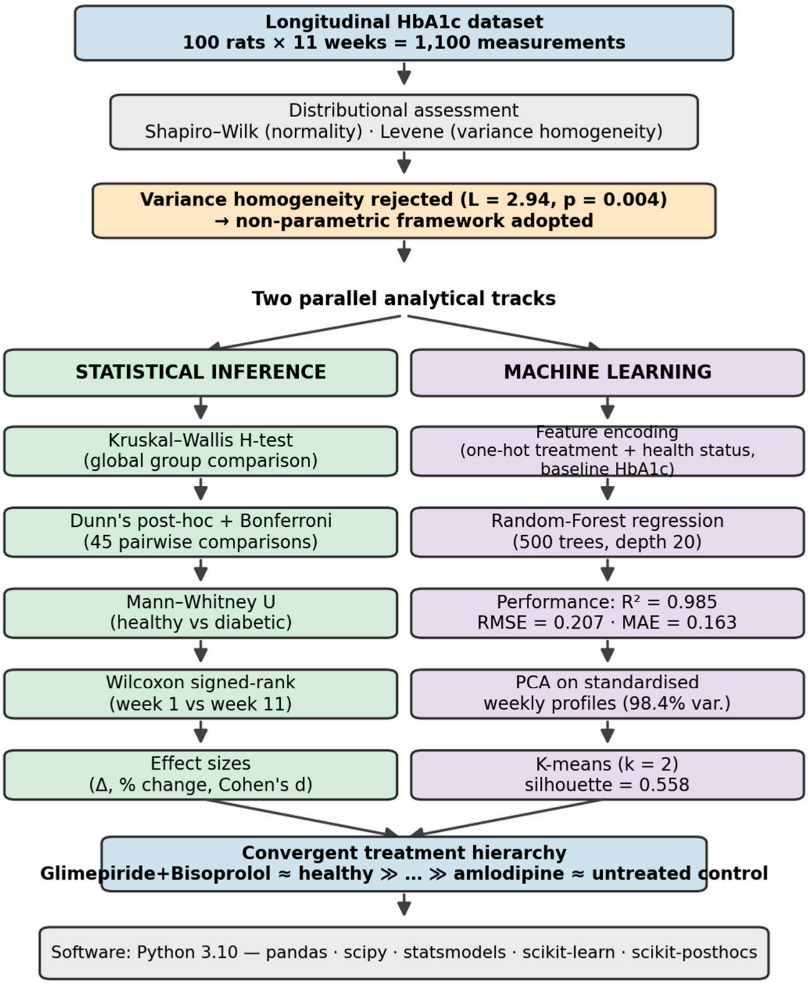

Figure S2. Overview of the data-processing and analysis workflow. The longitudinal HbA1c dataset underwent distributional assessment; because variance homogeneity was rejected, a non-parametric statistical framework was adopted in parallel with a machine-learning track (Random-Forest regression and PCA/K-means clustering). The two tracks converged on the same treatment hierarchy.
